# From “spleen governing muscle” to gut microbiota: mechanisms of sarcopenic obesity in perimenopausal women

**DOI:** 10.3389/fmicb.2026.1817839

**Published:** 2026-05-18

**Authors:** Yuanyuan Jin, Jianfang Zhu, Dan Liao, Xiaoqing Jin

**Affiliations:** 1 Department of Acupuncture and Moxibustion, Zhejiang Hospital, Hangzhou, China; 2 Department of Nutrition, Zhejiang Hospital, Hangzhou, China

**Keywords:** gut microbiota, multi-modal intervention, perimenopause, sarcopenic obesity, spleen governing muscle

## Abstract

**Background:**

Sarcopenic obesity (SO) in perimenopausal women—characterized by the paradoxical coexistence of skeletal muscle loss and visceral adiposity—represents a complex metabolic challenge that conventional estrogen replacement therapies have failed to fully address. Emerging evidence from traditional Chinese medicine (TCM) suggests that the “spleen governing muscle” theory may offer a unique lens through which to understand this condition, with the gut microbiota serving as a critical mechanistic link between spleen deficiency and musculoskeletal deterioration.

**Objective:**

To systematically review the mechanisms linking spleen deficiency, gut microbiota dysbiosis, and sarcopenic obesity risk in perimenopausal women, integrating TCM theory with modern biomedical evidence.

**Methods:**

We synthesized findings from clinical observational studies, randomized controlled trials (RCTs), and mechanistic investigations examining the “spleen-gut-muscle” axis, with particular attention to studies employing 16S rRNA sequencing, metabolomics, and multi-omics approaches.

**Results:**

Spleen deficiency—manifesting as impaired transformation and transportation of nutrients—correlates with characteristic alterations in gut microbiota composition, including reduced diversity, elevated *Firmicutes*/Bacteroidetes ratios, and depletion of short-chain fatty acid (SCFA)-producing taxa. These microbial changes propagate systemic inflammation through lipopolysaccharide (LPS)-TLR4 signaling, activate muscle protein degradation *via MuRF1* upregulation, and compromise insulin sensitivity through diminished GLP-1 secretion. Notably, TCM interventions—including herbal formulations (Sijunzi decoction, Buzhong Yiqi decoction), acupuncture, moxibustion, catgut embedding, and tuina massage—demonstrate potential to restore microbial homeostasis, increase SCFA production, and improve muscle mass and function. However, the evidence quality remains modest, with methodological limitations including inadequate blinding, small sample sizes, and short follow-up durations.

**Conclusion:**

The “spleen-gut-muscle” axis represents a promising therapeutic target for perimenopausal sarcopenic obesity, bridging TCM theory with modern microbiome science. Nevertheless, current evidence is predominantly associative rather than causally definitive, derived largely from small-scale trials and preclinical models. Rigorous, large-scale RCTs with standardized TCM protocols, multi-omics endpoints, and extended follow-up are essential to establish efficacy and safety before microbiota-based TCM interventions can be recommended as adjunctive or standalone therapies.

## 1 Introduction

Sarcopenic obesity (SO) has emerged as a critical yet underrecognized metabolic syndrome in the perimenopausal transition—a window of physiological vulnerability marked by profound hormonal fluctuations and accelerated body composition deterioration. Unlike simple sarcopenia or isolated obesity, SO presents a particularly insidious phenotype wherein the loss of skeletal muscle mass and function occurs concurrently with abnormal accumulation of visceral and intermuscular adiposity (Collins et al., 2019; Javed et al., 2019). This paradoxical combination appears to generate metabolic consequences that substantially exceed the additive risks of either condition alone, predisposing affected women to falls, fractures, functional disability, type 2 diabetes, and cardiovascular mortality (Collins et al., 2019).

Systematic reviews and meta-analyses report that the prevalence of sarcopenic obesity ranges from approximately 15–20% in individuals over 65 years, with higher estimates of 35% observed among obese populations (Cruz-Jentoft et al., 2010; Prado et al., 2012). However, these prevalence estimates vary substantially depending on the diagnostic criteria employed (EWGSOP1 vs. EWGSOP2 vs. AWGS), body composition assessment methods (DXA vs. BIA), and population characteristics, limiting direct comparability across studies. Perimenopausal women represent a high-risk group due to ovarian functional decline and consequent estrogen level fluctuations. Cross-sectional surveys indicate that the prevalence of sarcopenic obesity among perimenopausal women aged 45–55 ranges from 25 to 30% (Greendale et al., 2019), though longitudinal data tracking the incidence during the menopausal transition remain limited.

For the purpose of this review, sarcopenic obesity (SO) is operationally defined according to the European Working Group on Sarcopenia in Older People 2 (EWGSOP2) criteria (Cruz-Jentoft et al., 2019): the coexistence of (1) low muscle strength (handgrip strength <16 kg for women or chair stand test >15 s for 5 rises), (2) low muscle quantity (appendicular skeletal muscle index [ASMI] < 5.5 kg/m^2^ for women DXA or <5.5 kg/m^2^ BIA), and (3) high adiposity (visceral adipose tissue >100 cm^2^ or waist circumference >88 cm, combined with body fat percentage >35% for women) (World Health Organization, 1995). Alternatively, studies employing Foundation for the National Institutes of Health (FNIH) criteria (ALM/BMI < 0.512 for women) were included when body composition data permitted cross-walk comparison (Studenski et al., 2014). The perimenopausal period is defined as the interval beginning with cycle irregularity and ending 12 months after the final menstrual period, typically characterized by serum estradiol levels fluctuating between 10 and 100 pg./mL and follicle-stimulating hormone (FSH) > 25 IU/L (Harlow et al., 2012).

The precipitous decline in circulating 17β-estradiol concentrations—typically falling from 100 to 250 pg./mL during reproductive years to below 10–20 pg./mL within 12–24 months of menopause—has long been considered the principal driver of this pathological process (Randolph et al., 2004). Estrogen deficiency attenuates anabolic signaling through the IGF-1/PI3K/AKT cascade while upregulating ubiquitin ligases including muscle ring finger protein 1 (*MuRF1*) and muscle atrophy F-box protein (*MAFbx*), thereby disrupting protein homeostasis in skeletal muscle (Pellegrino et al., 2022). Simultaneously, the withdrawal of estrogen’s suppressive effects on adipose tissue promotes visceral fat deposition and chronic low-grade inflammation (Abildgaard et al., 2021).

Yet the clinical application of hormone replacement therapy (HRT) has demonstrated heterogeneous outcomes, with meta-analytical evidence suggesting only modest preservation of lean mass (approximately 0.06 kg reduction in loss) and potential cardiovascular complications that have tempered enthusiasm for widespread adoption (Javed et al., 2019; Armeni et al., 2021). This therapeutic inconsistency strongly implies the involvement of additional regulatory pathways beyond simple estrogen replacement—pathways that may be accessible through alternative medical systems, particularly traditional Chinese medicine (TCM).

The TCM concept of “spleen governing muscle”, first articulated in the Yellow Emperor’s Inner Canon (Huangdi Neijing) over two millennia ago, posits that the spleen serves as the foundation of postnatal existence, transforming food and drink into qi and blood to nourish the musculature (Tao et al., 2024). Spleen deficiency— manifesting as impaired transformation and transportation— theoretically leads to muscle wasting through failure to deliver adequate nutritional support, while simultaneously engendering dampness and phlegm accumulation that manifests as obesity (Tao et al., 2024; Ran et al., 2021). This dual pathology strikingly mirrors the modern conception of sarcopenic obesity.

Crucially, contemporary research has illuminated a potential biological substrate for this ancient theory: the gut microbiota. Increasingly conceptualized as a “virtual metabolic organ,” the intestinal microbiome possesses a metabolic repertoire vastly exceeding the host’s genomic capabilities, generating short-chain fatty acids (SCFAs), modifying bile acid profiles, and modulating inflammatory cascades that directly affect muscle and adipose tissue metabolism (Liu et al., 2021; Kwa et al., 2016). The “spleen-gut-muscle” axis—an modern extrapolation of classical TCM theory—suggests that spleen deficiency may manifest as characteristic alterations in gut microbiota composition, which in turn propagate systemic metabolic dysfunction (Tao et al., 2024; Chen et al., 2019).

This narrative review synthesizes evidence from PubMed, Embase, Cochrane, CNKI, and Wanfang (inception–March 2026) using MeSH terms and keywords for sarcopenic obesity, perimenopause, gut microbiota, and TCM. Inclusion: peer-reviewed clinical trials, mechanistic studies, and meta-analyses (English/Chinese). Exclusion: case reports, conference abstracts without data, preprints, and studies with poorly defined diagnostic criteria. Sarcopenic obesity was defined as the coexistence of sarcopenia (per EWGSOP2 criteria: handgrip strength <16 kg for women and/or ASMI <5.5 kg/m^2^) (Cruz-Jentoft et al., 2019) and obesity (body fat percentage >35% for women, per WHO criteria) (World Health Organization, 1995). Spleen deficiency was operationalized according to the according to the “Expert Consensus on TCM Diagnosis and Treatment of Spleen Deficiency Syndrome (2017)” (Professional Committee on Spleen and Stomach Diseases, China Association of Chinese Medicine) (Zhang et al., 2017), characterized by: (1) major symptoms (≥2 of: reduced appetite, postprandial abdominal distension, loose stools); (2) minor symptoms (≥1 of: fatigue, sallow complexion, weakness); and (3) tongue/pulse signs (pale tongue with tooth marks, weak pulse). Systematic review quality was assessed via AMSTAR 2 and PRISMA 2020.

This comprehensive review systematically examines the existing evidence regarding the putative influence of the “spleen-gut-muscle” axis on sarcopenic obesity risk in perimenopausal women, critically evaluates the underlying molecular mechanisms and methodological limitations of current research, and explores potential translational applications of multi-modal TCM interventions. We pay particular attention to the quality of available evidence, acknowledging that much of the current literature remains preliminary and subject to significant methodological constraints.

## 2 Theoretical foundations: from “spleen governing muscle” to the “spleen-gut-muscle” axis

### 2.1 Classical TCM theory and modern interpretation

The concept that the spleen governs muscle is deeply rooted in classical Chinese medical texts. The Plain Questions (Suwen) states unequivocally: “The spleen governs the muscles of the entire body”, while the Divine Pivot (Lingshu) elaborates that “when the spleen is diseased, the body feels heavy and the muscles become flaccid” (Tao et al., 2024). The General Treatise on the Etiology and Symptomatology of Diseases (Zhubing Yuanhou Lun) further explains that “when spleen-stomach qi is harmonious, it can circulate fluids to nourish the muscles; when spleen-stomach is deficient and weak, unable to transform and transport grains, then qi and blood are diminished, unable to irrigate the physical form, hence the muscles are not plump but withered and thin” (Tao et al., 2024).

This classical formulation bears striking resemblance to modern understanding of sarcopenia. The “spleen” in TCM encompasses not merely the anatomical organ but a functional system governing digestion, absorption, and distribution of nutrients—processes now understood to involve the gastrointestinal tract, enteric nervous system, and gut microbiota (Tao et al., 2024; Chen et al., 2019). The “muscles” in TCM similarly extend beyond skeletal muscle to include adipose tissue and connective tissue, with the “spleen governing muscle” theory thus implicating regulatory mechanisms that influence both muscle mass and fat distribution (Tao et al., 2024).

The Ming dynasty physician Li Ting, in his Introduction to Medicine (Yixue Rum), first proposed that “the spleen and small intestine are internally-externally related”, emphasizing the coordinated function of these organs in absorbing and distributing refined nutrients (Chen et al., 2019). Modern research has extended this concept to formulate the “spleen-gut-muscle” model, wherein muscle functional status is intimately connected with the stomach’s reception, the small intestine’s transformation, the large intestine’s transmission, and the spleen’s transportation functions (Tao et al., 2024; Chen et al., 2019). At the microscopic level, skeletal muscle cell apoptosis and mitochondrial structural damage are now recognized as the cellular manifestations of “spleen governing muscle” dysfunction (Tao et al., 2024).

### 2.2 Spleen deficiency as a clinical phenotype

Contemporary clinical investigations have validated the association between spleen deficiency patterns and sarcopenic obesity. A study of elderly patients with sarcopenia demonstrated that over 80% exhibited spleen deficiency patterns, characterized by reduced serum D-xylose absorption rates (indicating impaired intestinal absorption), diminished appetite, fatigue, and indigestion (Tao et al., 2024; Chen et al., 2019). Notably, these patients also displayed reduced diversity in gut microbiota composition, suggesting that spleen deficiency may have a discernible biological signature (Tao et al., 2024).

In the context of perimenopausal women, spleen deficiency often manifests as a complex pattern of “spleen deficiency with dampness” or “qi deficiency with blood stasis”—precisely the combination that would theoretically produce both muscle wasting (from inadequate nutritional support) and obesity (from impaired fluid metabolism) (Ran et al., 2021). The perimenopausal decline in estrogen exacerbates this process, as estrogen normally enhances spleen-stomach function and promotes efficient nutrient utilization (Randolph et al., 2004; Abildgaard et al., 2021).

**Figure 1:**
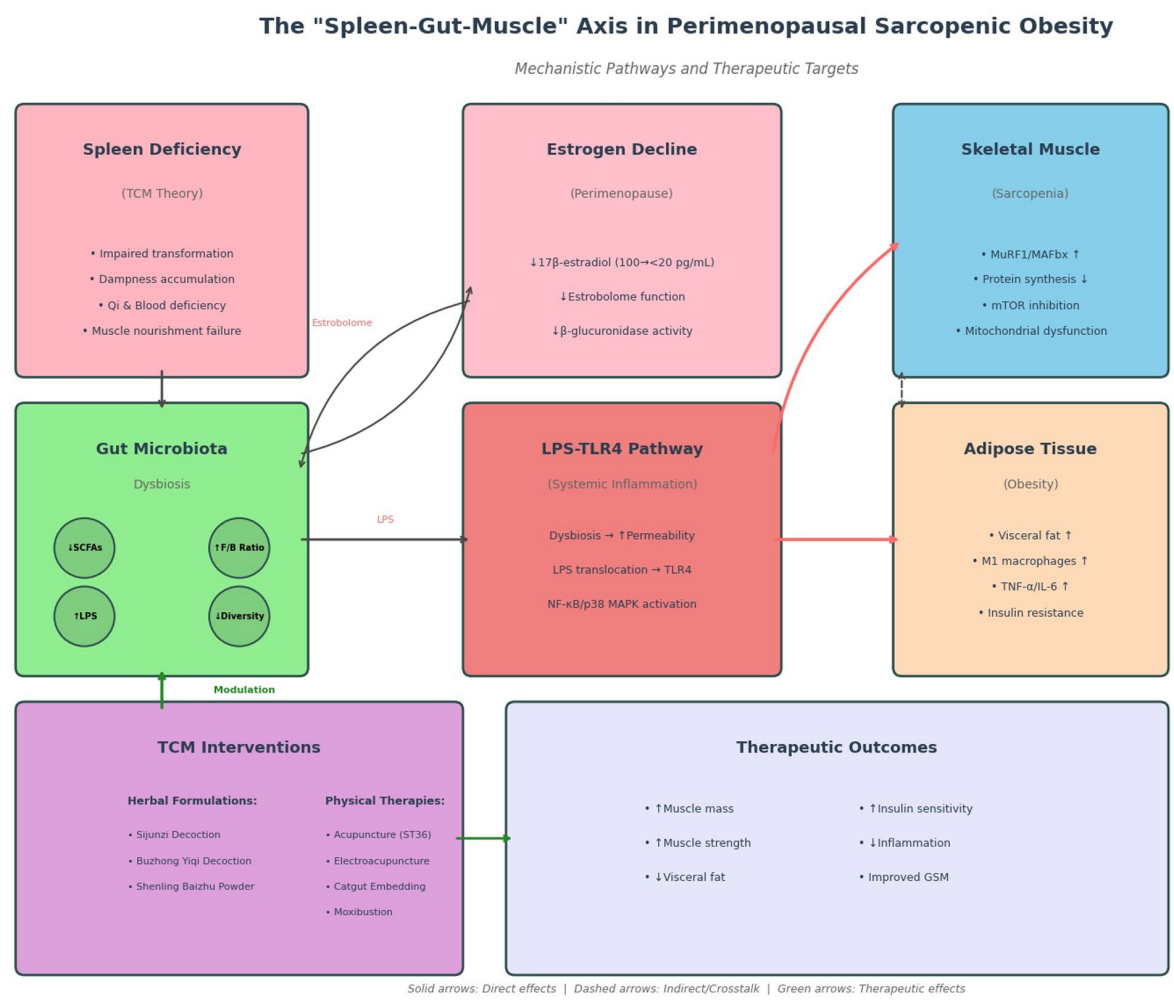
Schematic illustration of mechanistic pathways linking spleen deficiency (TCM pattern), perimenopausal estrogen decline, gut microbiota dysbiosis, and tissue dysfunction. Spleen deficiency and estrogen deficiency drive gut dysbiosis (reduced SCFAs, elevated F/B ratio, increased LPS), which activates the LPS-TLR4 inflammatory pathway, leading to skeletal muscle atrophy (MuRF1/MAFbx upregulation) and visceral adipose expansion. TCM interventions (herbal formulations, acupuncture, moxibustion, catgut embedding) modulate these pathways to restore microbial homeostasis and improve metabolic outcomes.

To visually integrate the classical TCM framework with modern mechanistic insights, Figure 1 illustrates the “Spleen-Gut-Muscle” axis, depicting how spleen deficiency and estrogen decline converge to drive gut dysbiosis, systemic inflammation, and subsequent tissue dysfunction in perimenopausal sarcopenic obesity.

## 3 Spleen deficiency and gut microbiota: evidence of association

### 3.1 Characteristic microbial alterations in spleen deficiency

The hypothesis that spleen deficiency correlates with specific gut microbiota alterations has gained empirical support from multiple studies employing 16S rRNA sequencing and metagenomic approaches. A study of patients with non-alcoholic fatty liver disease (NAFLD) stratified by TCM pattern differentiation revealed that those with spleen deficiency exhibited distinct microbial profiles compared to non-deficient controls: *Collinsella* and *Rhizopus* were significantly enriched, while *Intestinimonas* was depleted (Lin et al., 2025). Concurrently, spleen deficiency patients demonstrated reduced dietary diversity, inadequate intake of antioxidant nutrients (carotenoids, folate), and elevated inflammatory dietary indices (Lin et al., 2025).

In a rat model of diarrhea-predominant irritable bowel syndrome (D-IBS) with liver depression and spleen deficiency pattern, 16S rRNA sequencing demonstrated significant alterations in species richness, *α*-diversity, and *β*-diversity compared to controls (Wang Y. et al., 2025). The spleen deficiency model exhibited increased *Bacteroidota* and reduced *Firmicutes* at the phylum level, with elevated abundance of potentially pathogenic *Bacteroidales* (Wang Y. et al., 2025). These findings suggest that spleen deficiency states—whether in humans or experimental animals—are accompanied by reproducible alterations in gut microbiota structure.

### 3.2 The estrogen-microbiota nexus in perimenopause

The perimenopausal transition introduces additional complexity to the spleen-gut-muscle axis through the precipitous decline in estrogen levels, which directly influences gut microbiota composition. Comparative metagenomic studies have demonstrated that postmenopausal women exhibit reduced microbial β-glucuronidase (gmGUS) abundance and diversity compared to premenopausal individuals, potentially impairing enterohepatic estrogen recirculation (Jin et al., 2026; Hu et al., 2023). The *Firmicutes*/Bacteroidetes (F/B) ratio, a frequently cited marker of metabolic health, increases in postmenopausal women and correlates positively with obesity indices (Jin et al., 2026; Peters et al., 2022).

Specific taxa demonstrate estrogen-sensitive abundance patterns: *Bacteroides*, *Lactobacillaceae* bacterium 1157FAA, and *Roseburia*—generally considered beneficial—are abundant in premenopausal women but depleted after menopause (Jin et al., 2026; Peters et al., 2022). Conversely, potentially pathogenic *Enterobacteriaceae* may expand in the estrogen-deficient environment (Jin et al., 2026). These alterations have functional consequences: reduced gmGUS activity impairs estrogen deconjugation and reabsorption, effectively lowering systemic estrogen availability; diminished SCFA-producing taxa compromise metabolic regulation; and increased LPS-producing bacteria promote systemic inflammation (Jin et al., 2026; Hu et al., 2023; Peters et al., 2022).

The bidirectional nature of this relationship warrants emphasis. Just as estrogen deficiency shapes microbiota composition, microbial alterations influence estrogen metabolism through the “estrobolome”—the aggregate of microbial genes capable of metabolizing estrogens (Hu et al., 2023; Pollet et al., 2017). This establishes a potentially self-amplifying cycle wherein menopause-associated dysbiosis exacerbates estrogen deficiency, which in turn promotes further microbial deterioration (Jin et al., 2026; Peters et al., 2022).

### 3.3 Mechanistic pathways: from dysbiosis to metabolic dysfunction

The gut microbiota influences host metabolism through multiple convergent pathways that are particularly relevant to sarcopenic obesity pathogenesis:

Short-Chain Fatty Acids (SCFAs) and Metabolic Regulation. SCFAs—primarily acetate, propionate, and butyrate—are produced through bacterial fermentation of dietary fiber and serve as critical signaling molecules. They activate G-protein-coupled receptors (GPR41/FFAR3, GPR43/FFAR2) and inhibit histone deacetylases (HDACs), thereby regulating energy metabolism, insulin sensitivity, and inflammatory responses (Liu et al., 2021; Chambers et al., 2019). Butyrate, in particular, enhances mitochondrial function through PGC-1*α* activation and promotes muscle protein synthesis *via* mTOR signaling (Chambers et al., 2019; Zhang X. et al., 2024). The depletion of butyrate-producing taxa (e.g., *Faecalibacterium prausnitzii*, *Roseburia*) in spleen deficiency and menopausal states thus has direct implications for muscle health (Lin et al., 2025; Jin et al., 2026).

Lipopolysaccharide (LPS) and Systemic Inflammation. Dysbiosis-associated increases in intestinal permeability (“leaky gut”) permit translocation of LPS into the systemic circulation, activating Toll-like receptor 4 (TLR4) signaling in multiple tissues (Doyle et al., 2011; Cani et al., 2007). In skeletal muscle, LPS-TLR4 activation upregulates ubiquitin ligases *MuRF1* and atrogin-1/*MAFbx*, driving protein degradation and muscle atrophy (Doyle et al., 2011). In adipose tissue, LPS promotes M1 macrophage polarization and secretion of pro-inflammatory cytokines including TNF-*α* and IL-6, which further impair insulin sensitivity and promote lipolysis (Cani et al., 2007; Alizadeh, 2022). These cytokines also directly inhibit muscle protein synthesis through suppression of the PI3K/AKT/mTOR pathway (Shi et al., 2025).

Bile Acid Metabolism. Gut microbiota modify primary bile acids into secondary forms that signal through TGR5 and FXR receptors to regulate lipid metabolism and energy expenditure (Collins et al., 2023). Estrogen deficiency-associated dysbiosis alters bile acid pool composition, potentially compromising metabolic homeostasis (Collins et al., 2023; Fuhrman et al., 2014).

**Figure 2:**
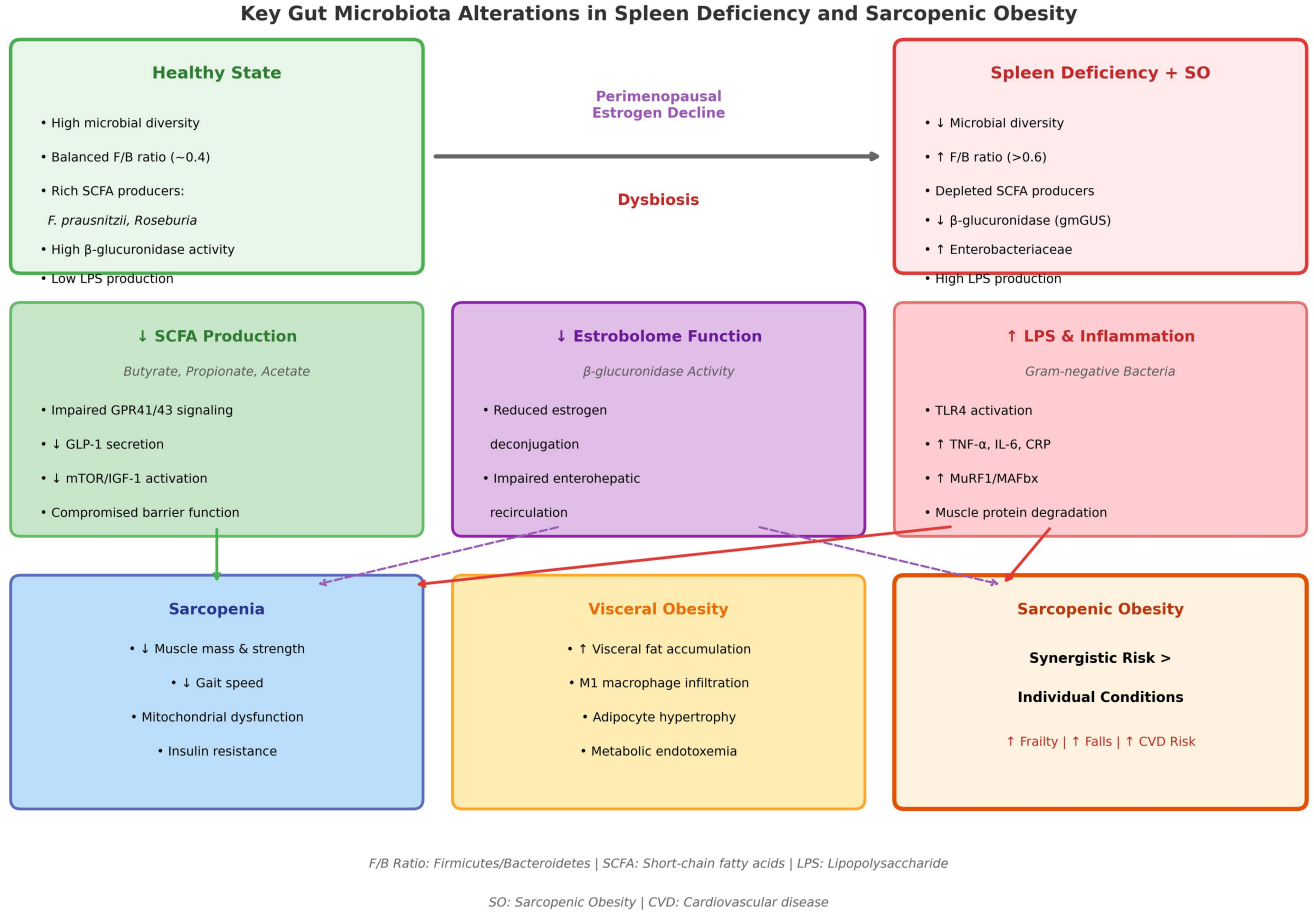
Comparative analysis of gut microbiota in healthy state versus spleen deficiency with sarcopenic obesity (SO). Perimenopausal estrogen decline drives dysbiosis: reduced diversity, elevated F/B ratio (>0.6), depleted SCFA producers, and increased LPS-producing bacteria. This triggers three pathways: (1) decreased SCFAs impairing GPR41/43 signaling and GLP-1 secretion; (2) Compromised estrobolome function reducing estrogen recirculation; and (3) LPS-TLR4 activation promoting systemic inflammation. Consequences include sarcopenia, visceral obesity, and synergistic SO risk.

These mechanistic alterations are summarized in Figure 2, which contrasts the healthy gut microbiota state with the dysbiotic profile characteristic of spleen deficiency and sarcopenic obesity, highlighting the three principal pathways—reduced SCFA production, compromised estrobolome function, and LPS-driven inflammation—that mediate disease progression.

## 4 Inflammatory mediators and muscle-adipose crosstalk

### 4.1 The central role of C-reactive protein and cytokines

While the gut microbiota provides the initiating stimulus, systemic inflammatory mediators serve as the proximate effectors of muscle and adipose tissue dysfunction in sarcopenic obesity. Recent evidence has elevated C-reactive protein (CRP) from a nonspecific marker to a causally implicated player in sarcopenia pathogenesis. A cross-sectional study of 207 patients demonstrated that CRP levels were significantly elevated in sarcopenic individuals and independently associated with reduced grip strength (R = −0.454), appendicular skeletal muscle mass (R = −0.426), and gait speed (R = −0.431) (Lin et al., 2024). Mendelian randomization analysis further supported a causal relationship between genetically predicted CRP levels and reduced muscle mass (Lin et al., 2024).

TNF-*α* and IL-6 remain the most extensively characterized cytokines in this context. TNF-α activates NF-κB signaling to upregulate *MuRF1* and atrogin-1 expression, promotes oxidative stress and mitochondrial dysfunction, and enhances myostatin signaling—creating a pro-catabolic environment in skeletal muscle (Alizadeh, 2022; Schaap et al., 2006). Longitudinal studies have confirmed that elevated TNF-α predicts accelerated muscle loss over 5-year follow-up periods (Schaap et al., 2006). IL-6, while classically considered a myokine with potential beneficial effects when released during exercise, demonstrates deleterious effects when chronically elevated in the context of systemic inflammation: it accelerates protein catabolism through JAK/STAT pathway activation and correlates with disability and functional decline (Alizadeh, 2022; Shi et al., 2025).

Critical Considerations. The inflammatory cascade is not unidirectionally deleterious. Acute IL-6 elevation during physical exercise, for instance, activates AMPK and promotes muscle hypertrophy—a reminder that context and kinetics matter (Alizadeh, 2022). Similarly, TNF-α participates in normal immune surveillance and tissue remodeling. The chronic, low-grade inflammation characteristic of spleen deficiency and menopausal dysbiosis appears to represent a pathological deviation from these physiological roles, but the precise thresholds distinguishing adaptive from maladaptive inflammation remain poorly defined (Alizadeh, 2022; Shi et al., 2025).

### 4.2 Insulin resistance and mitochondrial dysfunction

The convergence of spleen deficiency, dysbiosis, and estrogen deficiency creates a perfect storm for insulin resistance. TNF-*α* and IL-6 impair insulin receptor substrate (IRS) phosphorylation, while reduced SCFA production diminishes GLP-1 secretion and compromises pancreatic *β*-cell function (Liu et al., 2021; Chambers et al., 2019; Alizadeh, 2022). Skeletal muscle, as the primary site of insulin-mediated glucose disposal, suffers doubly: from the direct effects of inflammatory cytokines on insulin signaling, and from the loss of muscle mass itself, which reduces overall glucose uptake capacity (Pellegrino et al., 2022; Alizadeh, 2022).

Mitochondrial dysfunction represents another critical node. Estrogen normally promotes mitochondrial biogenesis and function through ERα-mediated PGC-1α activation; its withdrawal compromises ATP generation, increases reactive oxygen species (ROS) production, and accelerates cellular apoptosis (Pellegrino et al., 2022; Park et al., 2017). The reduction in butyrate-producing bacteria further impairs mitochondrial function, as butyrate serves as a preferred fuel for colonocytes and enhances oxidative metabolism in peripheral tissues (Chambers et al., 2019; Zhang X. et al., 2024).

## 5 Multi-modal TCM interventions: from herbs to acupuncture

### 5.1 Herbal formulations: restoring spleen function

Sijunzi Decoction—the foundational formula for spleen qi deficiency comprising Ginseng, Atractylodes, Poria, and Glycyrrhiza—has demonstrated efficacy in improving muscle mass and function in randomized trials. A study of 100 elderly sarcopenic patients found that modified Sijunzi decoction combined with resistance band training for 8 weeks significantly improved grip strength (23.75 ± 3.86 kg vs. 20.05 ± 3.69 kg in controls), appendicular skeletal muscle mass index (ASMI) (6.53 ± 0.41 kg/m^2^ vs. 6.26 ± 0.32 kg/m^2^), and Short Physical Performance Battery (SPPB) scores (9.54 ± 1.85 vs. 8.14 ± 1.96) (Wang et al., 2022).

Buzhong Yiqi Decoction—indicated for spleen qi sinking patterns—has shown particular promise in modulating inflammatory profiles. A randomized trial demonstrated that modified Buzhong Yiqi decoction combined with conventional intervention for 2 months significantly reduced serum IL-6 and TNF-α levels without adverse effects on hematological or hepatic/renal parameters (Chen et al., 2021). The formula’s monarch herb, *Astragalus membranaceus* (Huangqi), contains *Astragalus* polysaccharides (APS) that have been extensively studied for their microbiota-modulating effects. *In vitro* fermentation models demonstrate that APS increases Bifidobacterium and Lactobacillus abundance while reducing *Escherichia-Shigella*; it significantly elevates propionate levels and partially restores acetate and butyrate (Zhang X. et al., 2024). In *db/db* mice, APS administration restored microbial community structure, increased *Akkermansia* and *Faecalibaculum* abundance, and enhanced fecal antioxidant metabolites including all-trans-retinoic acid and thiamine (Song et al., 2022).

Shenling Baizhu Powder—indicated for spleen deficiency with dampness—regulates intestinal microecology and modulates gastrointestinal hormones including ghrelin and obestatin, enhancing appetite and gastrointestinal motility (Jiang et al., 2024). A trial in elderly type 2 diabetes patients with sarcopenia demonstrated that Shenling Baizhu powder combined with exercise improved glycemic control, grip strength, and gait speed while reducing fall risk (Jiang et al., 2024).

### 5.2 Acupuncture and electroacupuncture: neuromodulation of muscle metabolism

Acupuncture stimulation at ST36—a he-sea point of the stomach meridian traditionally indicated for strengthening the spleen and benefiting qi—has been shown in preclinical studies to upregulate serum IGF-1 levels, activate muscle satellite cells, and promote muscle protein synthesis (Li et al., 2025). In clinical trials, twice-weekly electroacupuncture at yangming meridian acupoints over 12 weeks significantly improved ASMI, grip strength, and body composition compared with nutritional therapy alone (Ma et al., 2023). Similarly, electroacupuncture combined with rehabilitation exercise training for 4 weeks significantly increased SPPB scores, walking distance, and muscle mass compared to exercise alone (Ling et al., 2022). However, not all studies have shown positive results; a Brazilian trial found no significant objective improvements in muscle mass or strength after 8 weeks of acupuncture, though participants reported subjective benefits (Soares et al., 2019).

Electroacupuncture (EA) introduces rhythmic electrical stimulation to traditional needling, potentially enhancing effects on muscle metabolism. In SAMP8 mice—a model of accelerated aging—EA combined with sulforaphane activated the AMPK/Sirt1/PGC-1*α* pathway, suppressed oxidative stress and inflammatory cytokines (IL-6, TNF-α), and repaired mitochondrial damage in skeletal muscle (Guo et al., 2024). EA alone also significantly inhibited muscle cell apoptosis and atrophy protein expression (Guo et al., 2024).

In type 2 diabetic rats, EA at ST36, SP6, and EX-B3 for 4 weeks significantly reduced fasting glucose, improved lipid profiles, and enhanced insulin sensitivity—effects mediated through AMPK/PGC-1α/TFAM pathway activation and increased GLUT4 expression in skeletal muscle (Luo et al., 2025). Notably, these beneficial effects were completely abolished by co-administration of the AMPK inhibitor Compound C, confirming the necessity of this pathway (Luo et al., 2025). EA combined with treadmill exercise demonstrated superior effects to either intervention alone in diet-induced obese rats, significantly upregulating PGC-1α, FNDC5 (irisin precursor), and phosphorylated AMPK in skeletal muscle compared with simple EA or simple treadmill exercise (*p* < 0.05) (Zhang et al., 2019).

### 5.3 Catgut embedding: sustained acupoint stimulation

Acupoint catgut embedding (ACE)—implanting absorbable sutures at acupoints to provide prolonged stimulation—offers practical advantages for chronic conditions requiring frequent treatment. A randomized trial in perimenopausal women with central obesity demonstrated that ACE significantly reduced body weight, BMI, waist circumference, and improved adiponectin levels (Jin et al., 2024). 16S rRNA sequencing revealed that ACE increased microbial diversity despite reducing species richness, with significant enrichment of *Kosakonia* and *Klebsiella*—taxa positively correlated with adiponectin and negatively correlated with body weight (Jin et al., 2024).

Mechanistic investigations suggest ACE may reduce adipocyte numbers through localized fat necrosis (Jin et al., 2024), improve leptin resistance through hypothalamic leptin receptor upregulation (Yan et al., 2012) and suppression of SOCS-3 expression (Yan et al., 2016), suppress inflammatory cytokine expression in adipose tissue (Guo et al., 2015), and activate Wnt/*β*-catenin signaling to inhibit lipogenesis (Chen L. et al., 2025). Systematic reviews confirm ACE’s efficacy in reducing body weight and waist circumference with acceptable safety profiles, though standardization of acupoint selection and treatment frequency remains incomplete (Guo et al., 2015; Yue et al., 2024).

### 5.4 Moxibustion and tuina: thermal and mechanical modulation

Moxibustion—thermal stimulation at acupoints—exerts warming and tonifying effects particularly suited for spleen yang deficiency patterns. While direct evidence for moxibustion in sarcopenic obesity remains limited, methodological assessments of moxibustion systematic reviews indicate moderate reporting quality and significant potential for chronic disease management (Chen R. et al., 2025). Proposed mechanisms include improved local circulation, TRPV1 channel activation, and gut microbiota modulation.

Tuina (Chinese therapeutic massage) provides direct mechanical stimulation to muscle tissue, improving local circulation, relieving fatigue, and potentially modulating muscle protein turnover (Guo et al., 2022). A systematic review of traditional Chinese exercise (including Yijinjing, Tai Chi, Baduanjin) and tuina for sarcopenia identified positive effects on gait speed, lower limb strength, and daily living activities, though evidence quality was limited by small sample sizes and lack of control groups (Guo et al., 2022). Tuina’s effects on gut microbiota remain essentially unexplored, representing a significant evidence gap.

**Figure 3:**
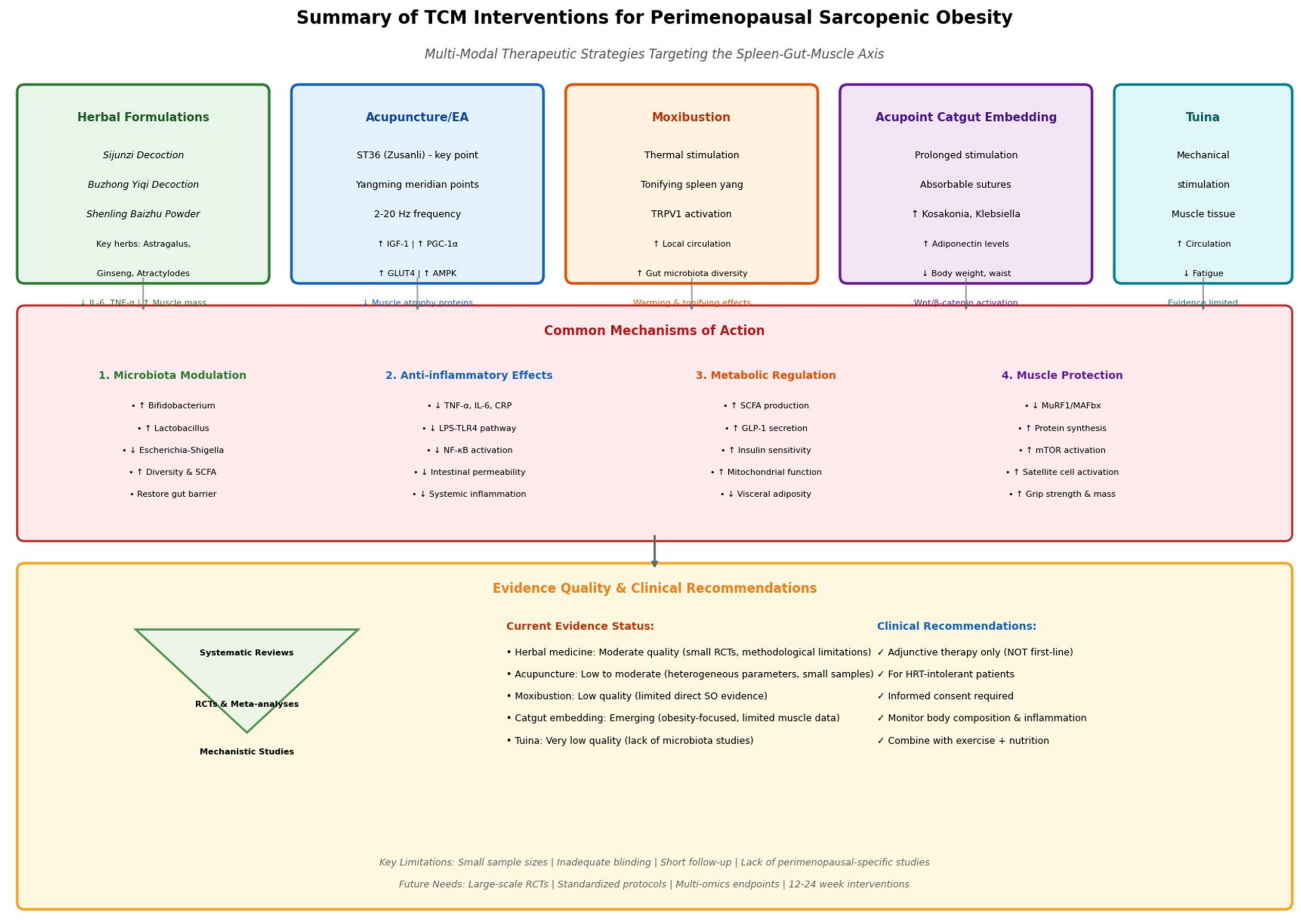
Overview of multi-modal TCM interventions targeting the spleen-gut-muscle axis. Five therapeutic approaches (herbal formulations, acupuncture/EA, moxibustion, catgut embedding, tuina) share common mechanisms: restoration of beneficial microbiota (*Bifidobacterium, Lactobacillus*), anti-inflammatory effects (↓ TNF-α, IL-6), metabolic regulation (↑ SCFAs, insulin sensitivity), and muscle protection (↓ MuRF1, ↑ protein synthesis). Evidence quality ranges from very low to moderate; current recommendations support use as adjunctive therapy with exercise and nutritional interventions.

Figure 3 provides a comprehensive summary of these multi-modal TCM interventions, outlining their mechanisms of action—from microbiota modulation and anti-inflammatory effects to metabolic regulation and muscle protection—and stratifying the current evidence quality for each therapeutic approach.

To provide a structured comparison of the clinical evidence evaluating TCM interventions for sarcopenic obesity and related metabolic disorders, Table 1 summarizes the key characteristics, principal findings, and methodological limitations of representative randomized controlled trials. This comparative overview reveals consistent trends toward improved muscle mass and metabolic markers across diverse TCM modalities, while highlighting pervasive design constraints—including inadequate blinding, small sample sizes, short intervention durations, and heterogeneous diagnostic criteria—that necessitate cautious interpretation and underscore the need for the critical appraisal presented in the following section.

**Table 1:** Summary of key clinical studies evaluating TCM interventions for sarcopenic obesity and related metabolic disorders.

Study (author, year)	Design	Population (*n*, age)	Diagnostic criteria	Intervention (duration)	Key findings	Major limitations
Wang et al. (2022)	Single-center RCT	Elderly with sarcopenia (*n* = 100, 65–85 yr)	AWGS 2019 (Handgrip < 18 kg, ASM < 5.7 kg/m^2^)	Modified Sijunzi decoction + resistance band vs. resistance band alone (8 weeks)	↑ Grip strength (23.75 vs. 20.05 kg); ↑ ASMI (6.53 vs. 6.26); ↑ SPPB scores	No blinding; no placebo control; short follow-up
Chen et al. (2021)	Single-center RCT	Elderly sarcopenia (*n* = 60)	EWGSOP2 criteria	Modified Buzhong Yiqi decoction + conventional care vs. conventional care (2 months)	↓ IL-6, TNF-α; improved muscle mass	Small sample; lack of sham control; no microbiome data
Ma et al. (2023)	RCT	Sarcopenic patients (*n* = 72, >65 yr)	AWGS 2019	Electroacupuncture (Yangming meridians) vs. nutritional therapy (12 weeks)	↑ ASMI, grip strength, body composition improvement	No sham acupuncture control; acupuncturist not blinded
Soares Mendes Damasceno et al. (2019)	RCT	Elderly with sarcopenia (*n* = 36)	EWGSOP2	Acupuncture vs. no intervention (8 weeks)	No significant objective improvement in muscle mass or strength; subjective benefits	Small sample; no sham control; high dropout rate
Jin et al. (2024)	Single-center RCT	Perimenopausal women with central obesity (*n* = 60, 45–55 yr)	IDF criteria (waist ≥ 80 cm)	Acupoint catgut embedding vs. sham embedding (12 weeks)	↑ Microbial diversity; ↑ Adiponectin; ↓ Body weight, WC	Short duration; no muscle strength endpoints; single center

ALM, appendicular lean mass; ASMI, appendicular skeletal muscle index; AWGS, Asian Working Group on Sarcopenia; EA, electroacupuncture; EWGSOP2, European Working Group on Sarcopenia in Older People 2; IDF, International Diabetes Federation; RCT, randomized controlled trial; SPPB, Short Physical Performance Battery; WC, waist circumference. This table illustrates the heterogeneity of diagnostic criteria currently employed in TCM intervention studies. While EWGSOP2 represents the primary standard adopted for this review, included studies utilized AWGS 2019 and IDF criteria, reflecting the lack of standardized diagnostic protocols in the field.

## 6 Critical appraisal: methodological quality and evidence limitations

### 6.1 Assessment of systematic reviews and meta-analyses

Applying AMSTAR 2, PRISMA 2020, and GRADE criteria to evaluate the methodological rigor of existing syntheses reveals substantial room for improvement. The majority of systematic reviews and meta-analyses examining TCM interventions for sarcopenia or related conditions demonstrate modest methodological quality, with frequent deficiencies in protocol registration, comprehensive literature searching, and risk of bias assessment.

**Figure 4:**
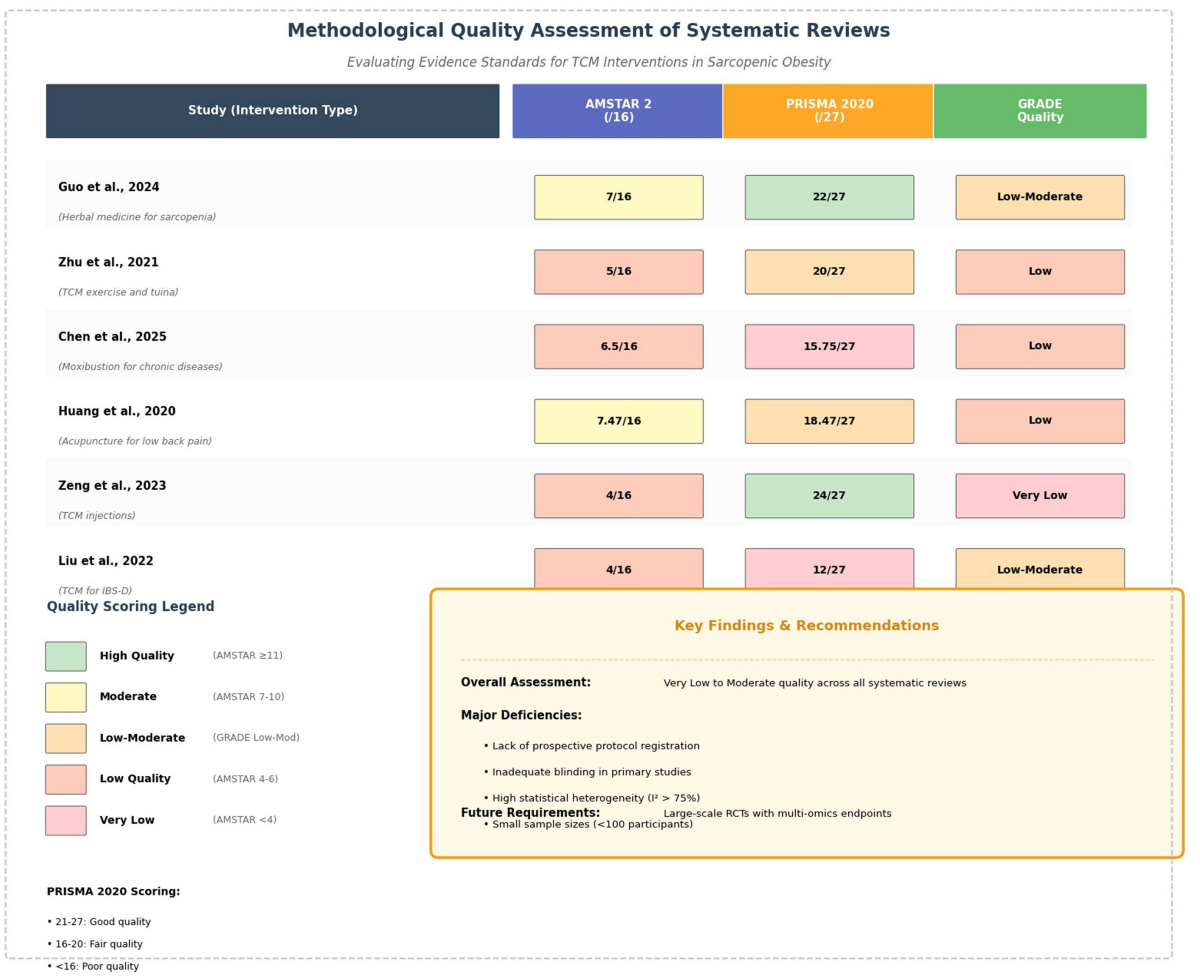
Quality evaluation of six systematic reviews using AMSTAR 2 (methodological rigor), PRISMA 2020 (reporting standards), and GRADE (evidence certainty) criteria. Overall quality ranges from very low to moderate across studies. Major deficiencies include lack of prospective protocol registration, inadequate blinding, high heterogeneity (I^2^ > 75%), and small sample sizes. Future research requires large-scale RCTs with standardized TCM protocols, multi-omics endpoints, and perimenopausal-specific populations.

The quality assessment results are visually presented in Figure 4, which evaluates six major systematic reviews using AM.

### 6.2 Major methodological deficiencies

Study Design Limitations. The TCM literature is characterized by several pervasive design flaws that compromise internal validity: (1) Inadequate blinding—the nature of herbal interventions and acupuncture makes double-blinding challenging, yet few studies employ rigorous sham controls or blinded outcome assessors (Guo et al., 2022; Zhang Y. et al., 2024); (2) Inappropriate control groups—many trials compare TCM interventions to “no treatment” or “routine care” rather than placebo or active comparators, making it impossible to distinguish specific from non-specific effects (Guo et al., 2022); (3) Lack of protocol registration—the majority of systematic reviews were not prospectively registered, introducing risks of selective reporting and outcome switching (Chen R. et al., 2025; Huang et al., 2020).

Population Specificity. Perhaps most critically, existing evidence fails to adequately address the perimenopausal population specifically. The vast majority of studies enroll elderly patients (≥60 years) without stratification by menopausal status, obscuring the unique pathophysiology of estrogen-deficient sarcopenic obesity (Guo et al., 2022; Zhang Y. et al., 2024). Gender-specific analyses are similarly lacking, despite recognized sex differences in muscle metabolism and microbiota composition (Jin et al., 2026; Peters et al., 2022).

Based on the very low to low quality of available evidence, TCM interventions for perimenopausal sarcopenic obesity should currently be considered as adjunctive therapies only, not replacements for conventional management (resistance training, protein supplementation, and where appropriate, hormone therapy). Clinicians should counsel patients regarding the preliminary nature of the evidence and the need for shared decision-making.

### 6.3 Contradictory findings and interpretive challenges

The literature contains notable contradictions that warrant careful consideration. SCFAs and HIF-1α signaling exemplify this complexity: while SCFAs generally suppress HIF-1α to prevent glycolytic fiber type switching in COPD-associated sarcopenia, HIF-1α also serves adaptive functions in hypoxic conditions, and its complete suppression might theoretically compromise cellular resilience (Tao et al., 2024; Zhang X. et al., 2024). The optimal SCFA concentration range remains undefined, and tissue-specific effects may differ from systemic administration.

The therapeutic potential of butyrate supplementation presents an additional pharmacokinetic paradox. Systemic sodium butyrate administration in OVX-induced obese mice ameliorates metabolic dysfunction through ERα-AMPK pathway activation in skeletal muscle (Fu et al., 2023). Conversely, microbiota-derived colonic butyrate acts primarily on local enteroendocrine cells, with only 5–10% reaching systemic circulation (Chambers et al., 2019). Oral butyrate undergoes rapid hepatic first-pass metabolism, compromising colonic delivery while achieving higher peripheral concentrations (Peng et al., 2023). Whether direct butyrate supplementation or microbiota-targeted interventions (prebiotics, TCM formulations) represent superior therapeutic strategies for sarcopenic obesity remains empirically unresolved, as tissue-specific concentration requirements and optimal delivery modalities are undefined (Zhao et al., 2018; Li et al., 2023).

The estrogen-microbiota relationship exhibits temporal and methodological discrepancies that complicate mechanistic interpretation. While several studies document significant gut microbiota alterations in OVX rodent models—including reduced Firmicutes, decreased diversity, and Proteobacteria expansion (Chen M. et al., 2025; Wang H. et al., 2025)—Sau et al. (2021). reported that short-term OVX (5 weeks) did not induce significant biodiversity changes despite pronounced metabolic dysfunction. This apparent contradiction likely reflects differential temporal dynamics: microbial compositional shifts may require >8–12 weeks to manifest following estrogen withdrawal, exceeding the duration employed in many mechanistic studies (Sau et al., 2021). Additionally, strain-specific variations in microbiota responses and methodological heterogeneity in 16S rRNA sequencing protocols contribute to inconsistent findings (Safari et al., 2020; Pranesh et al., 2025). The acute, complete estrogen ablation in OVX models fundamentally differs from the gradual, heterogeneous human menopausal transition, complicating direct translational extrapolation (Jin et al., 2026; Peters et al., 2022).

Electroacupuncture frequency parameters present critical translational limitations from preclinical to clinical application. While 2 Hz and 100 Hz stimulation demonstrate distinct neurochemical effects in rodent models—promoting enkephalin versus dynorphin release, respectively (Cheng et al., 2012)—these findings derive predominantly from animal studies with fundamentally different neurophysiological characteristics compared to humans. Optimal EA parameters for muscle metabolism specifically remain undefined in human trials; existing sarcopenia studies have employed heterogeneous frequency protocols (2–20 Hz) without systematic dose–response optimization (Ma et al., 2023; Ling et al., 2022; Guo et al., 2024). Individual variability in skin impedance, subcutaneous adipose tissue thickness, and peripheral nerve sensitivity substantially influences effective current delivery, yet standardized intensity calibration methods remain underdeveloped (He et al., 2025; Li et al., 2021). The absence of sham-controlled, frequency-stratified trials in perimenopausal women specifically—who may exhibit altered pain sensitivity and autonomic tone—represents a critical evidence gap (Ma et al., 2023; Ling et al., 2022; Soares et al., 2019).

Probiotic versus herbal approaches present another tension. Direct supplementation with specific strains (Lactobacillus, Bifidobacterium) has yielded inconsistent results in muscle function trials, while complex herbal formulations appear more consistently effective—yet the mechanisms are correspondingly more difficult to disentangle (Zhang X. et al., 2024; Song et al., 2022; Zhang Y. et al., 2024). Whether the “whole system” approach of TCM offers genuine synergistic advantages over isolated probiotics, or merely introduces confounding variability, remains unresolved.

## 7 Future directions and translational potential

### 7.1 Toward precision medicine: stratification and biomarkers

The future of TCM-based microbiota intervention lies in precision medicine approaches that acknowledge individual heterogeneity. “Pattern-microbiota” stratification—matching TCM syndromes (spleen qi deficiency, spleen yang deficiency, spleen deficiency with dampness) to specific microbial signatures—could enhance treatment specificity (Lin et al., 2025; Wang Y. et al., 2025). Biomarker-guided therapy, using baseline CRP, SCFA profiles, or specific taxa abundances to predict response, may improve efficiency and reduce trial-and-error prescribing (Lin et al., 2024; Jin et al., 2024).

Multi-omics integration offers a pathway to mechanistic clarity. Combining 16S rRNA or metagenomic sequencing with metabolomics (serum and muscle SCFAs, bile acids, tryptophan metabolites), proteomics (inflammatory mediators, muscle protein turnover markers), and transcriptomics (muscle atrophy gene expression) would enable construction of comprehensive “spleen-gut-muscle” interaction networks (Wang Y. et al., 2025; Zhang X. et al., 2024). Such approaches could identify which microbial functions are necessary and sufficient for muscle protection, guiding targeted interventions.

### 7.2 Multi-modal integration and clinical implementation

Given the multifactorial nature of perimenopausal sarcopenic obesity, multi-modal interventions combining herbal medicine, acupuncture, exercise, and dietary modification likely offer the greatest therapeutic potential. The synergistic effects observed with EA combined with exercise (Zhang et al., 2019) support this approach, though optimal combination protocols remain to be defined through factorial trial designs.

Technology-enabled delivery may overcome adherence barriers. Wearable devices for remote monitoring of physical activity, smart scales for body composition tracking, and telemedicine-based TCM consultation could enhance long-term engagement—critical given that microbiota modifications and muscle adaptations require sustained intervention (Guo et al., 2022).

### 7.3 Policy and regulatory pathways

Translation from research to clinical practice requires standardized TCM protocols developed through consensus processes and validated in rigorous RCTs. The development of hospital preparations or functional foods based on proven formulations (e.g., modified Sijunzi or Buzhong Yiqi decoctions) could bridge the gap between individualized prescribing and scalable production (Chen et al., 2021; Jiang et al., 2024).

Multidisciplinary care models integrating endocrinology, gynecology, rehabilitation medicine, nutrition, and TCM are essential for managing the complexity of perimenopausal metabolic disorders (Collins et al., 2019; Javed et al., 2019). Such models should emphasize shared decision-making, with microbiota-based TCM interventions positioned as adjunctive options for patients unwilling or unable to use conventional HRT, pending further evidence (Armeni et al., 2021; Jin et al., 2026).

## 8 Conclusion

The “spleen governing muscle” theory, reinterpreted through the lens of modern microbiome science, offers a coherent framework for understanding perimenopausal sarcopenic obesity that bridges classical TCM concepts with contemporary biomedical mechanisms. Spleen deficiency manifests as characteristic gut microbiota alterations—reduced diversity, elevated F/B ratios, depleted SCFA producers—that propagate systemic inflammation, insulin resistance, and muscle protein degradation. TCM interventions including herbal formulations, acupuncture, moxibustion, and catgut embedding demonstrate potential to restore microbial homeostasis and improve muscle outcomes, yet the evidentiary base remains preliminary.

Current limitations are substantial: methodological quality is modest, population specificity is lacking, mechanistic studies are predominantly associative, and intervention standardization is incomplete. Rigorous, large-scale randomized trials with standardized TCM protocols, multi-omics endpoints, extended follow-up, and specific focus on perimenopausal women are essential to establish whether microbiota-based TCM interventions can be recommended as adjunctive or standalone therapies for this increasingly prevalent and debilitating condition.
